# PSGpower: A MATLAB toolbox for analyzing sleep EEG data

**DOI:** 10.1016/j.softx.2025.102076

**Published:** 2025-02-01

**Authors:** Ahren B. Fitzroy, Rebecca M.C. Spencer

**Affiliations:** University of Massachusetts, Amherst, USA

**Keywords:** Sleep, EEG, Polysomnography, Signal processing, Neuroscience, Brain activity

## Abstract

Sleep science has seen a surge in discoveries fueled by enhanced data processing approaches to sleep physiology recordings. PSGpower is a MATLAB toolbox designed to make these processing steps more efficient and standardized. PSGpower imports sleep polysomnography data recorded using legacy and modern acquisition systems, and sleep-staged using a variety of software packages, for processing in a number of microstructure analysis workflows. Workflows include existing algorithms from EEGLAB and FieldTrip and custom algorithms. PSGpower is extensible, and new workflows can be added that take advantage of the common data importing, sleep stage marking, and preprocessing code.

**Table:** Metadata

Nr	Code metadata description	*Please fill in this column*
C1	Current code version	v1.0.0
C2	Permanent link to code/repository used for this code version	https://github.com/afitzroy/psgpower
C3	Permanent link to reproducible capsule	N/A
C4	Legal code license	GPLv3
C5	Code versioning system used	Git
C6	Software code languages, tools and services used	MATLAB
C7	Compilation requirements, operating environments and dependencies	MATLAB (≥ v2018a), EEGLAB v13.6.5b with plugins ERPLAB v6.1.4, BioSig, bva-io, and Fieldtrip-lite
C8	If available, link to developer documentation/manual	https://github.com/afitzroy/psgpower/wiki
C9	Support email for questions	ahren.fitzroy@gmail.com

## 1. Motivation and significance

Several high-quality open source toolboxes exist for analyzing electroencephalogram (EEG) data, including EEGLAB [1], ERPLAB [14], FieldTrip [17], Brainstorm [20], MNE [8], and SPM [7]. However, although these toolboxes are able to analyze EEG data collected from both waking and sleeping participants, these toolboxes are not optimized for the analysis of EEG data recorded during sleep. A primary issue is that sleep EEG data are routinely classified into discrete sleep stages at regular intervals as an initial analysis step, forming a second time axis (“stage time”) often of essential analytical interest instead of, or in complement to, chronological time. While it is possible to annotate sleep stages and use those annotations to guide analyses in the currently available EEG software packages, this process requires several manual steps of epoching and reconcatenation to perform common simple sleep EEG analysis tasks, such as isolating non-rapid eye movement (NREM) stage 3 sleep for spectral analysis. Moreover, existing EEG analysis toolboxes are not capable of automatically importing sleep stage notations created using clinical or open-source sleep EEG software, so stage notations need to be manually converted into a format acceptable by the chosen toolbox before they can be used to inform analyses.

**Fig. 1.**
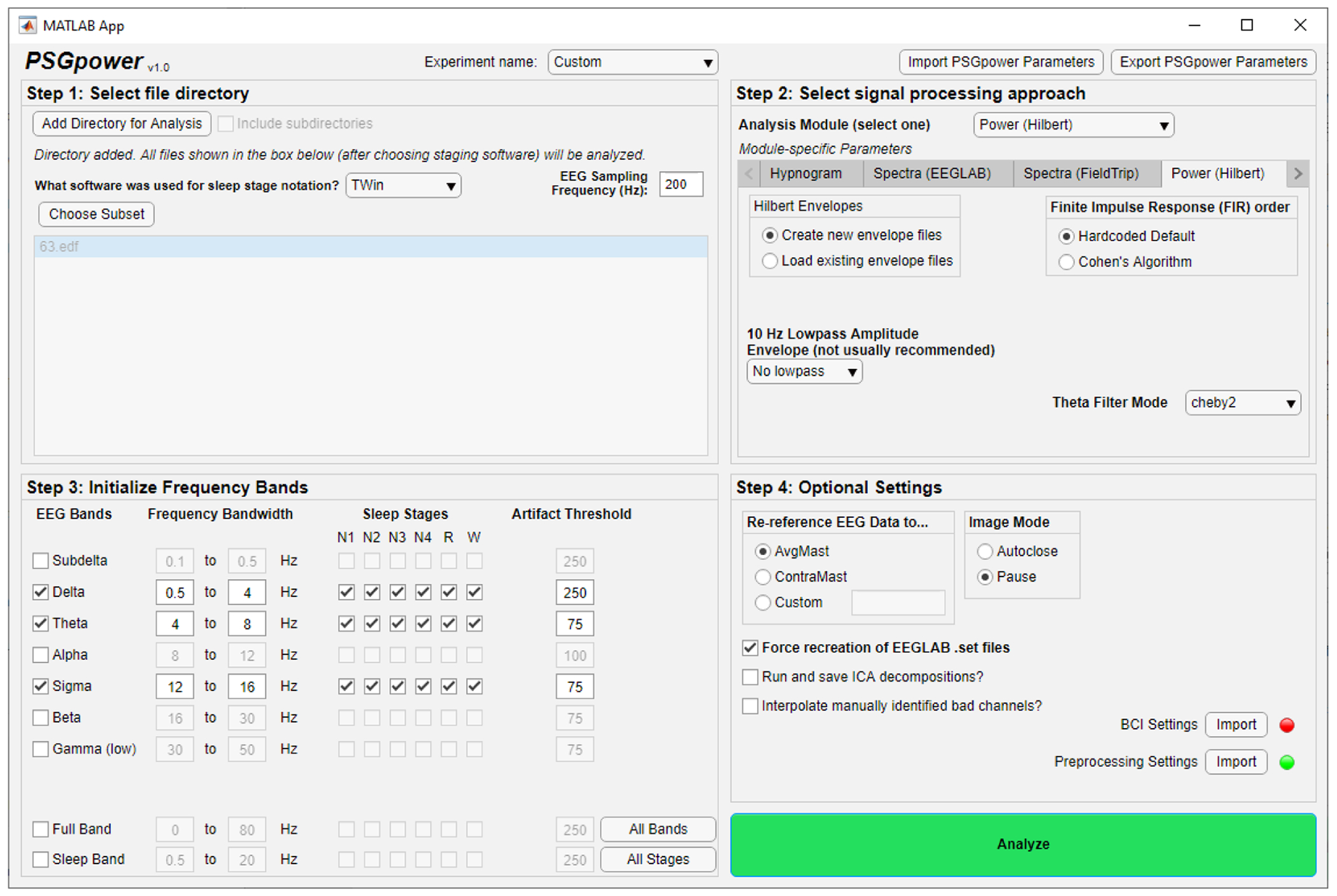
PSGpower GUI. The GUI is split into four Steps, which appear sequentially as the user populates options. The GUI dynamically updates the options presented in later Steps based on the choices in earlier Steps.

PSGpower aims to solve these issues by considering sleep stage notation metadata throughout the analysis process. PSGpower automatically imports stage notations from multiple specialized sleep EEG software output formats and converts them to a common EEGLAB-based tag marker structure, then allows simple specification of stage time restrictions in a graphical user interface (GUI; Fig. 1) prior to running analyses. This facilitates performing analyses in EEGLAB, FieldTrip, or custom algorithms that flexibly consider stage time, without requiring extensive coding experience of the user. Importantly, PSGpower is built to be extensible, allowing the easy addition of stage-time aware analysis “modules” implementing any desired custom MATLAB workflow that accepts EEGLAB-format sleep EEG data with stage notation metadata attached. Further, import functions for additional EEG recording systems or stage notation software can be easily added as required. This common analysis framework has benefitted researchers in our lab, and we believe it would be of utility to the broader community of sleep EEG/polysomnography (PSG) researchers as well.

Thus far our lab has implemented stage-time aware modules to quantify and compare spectral amplitude using cluster-based permutation analyses, to quantify and compare time-domain amplitude using the filter-Hilbert method or wavelet-based decomposition, and to analyze the temporal and topographical dynamics of multichannel amplitude envelopes. These modules have been applied by multiple researchers in our group, resulting in publications based on developmental [3,12,13,16], young adult [9–11,18], and aging [4–6] population sleep EEG data. Additionally, we have wrapped the code underlying a sleep spindle detection algorithm previously published by another research group [2] as a module, demonstrating the easy extensibility of PSGpower; we believe this provides a useful solution for standardizing custom processing algorithms implemented across sleep physiology research groups, enhancing efficiency of research, and thus facilitating discoveries in sleep science.

## 2. Software description

### 2.1. Software architecture

PSGpower is a MATLAB application built using the AppDesigner framework, and can be run directly within MATLAB or installed into the MATLAB Apps tab. Once launched, the user uses a GUI (Fig. 1) to select their EEG data files, indicate the sleep stage notation metadata format, select and configure an analysis module, indicate stage time and/or frequency band processing restrictions, set general processing options, and launch the analysis module. Once an analysis module is launched, dialogs may guide the user through additional run-time choices, then the graphical and/or statistical output of the module is displayed on screen and/or saved to disk.

The PSGpower GUI is broadly organized into four “Steps”, which are designed to guide the user sequentially through module selection and configuration. In Step 1, the user selects the directory containing their sleep EEG recordings and associated stage notation files, provides basic information about these files, and can flexibly select a subset of the files available in the specified folder to process. In Step 2, the user selects an analysis module from the dropdown list or tab group headings, then configures processing and analysis parameters specific to that module. In Step 3, the user indicates which frequency bands the chosen analysis module should be looped over, which sleep stages should be included within each frequency band, and what artifact detection thresholds should be used for each frequency band. In Step 4, the user chooses general preprocessing settings including how to re-reference the EEG data, whether or not to perform bad channel interpolation (BCI) and specifying which channels to interpolate if so, and whether or not to perform an independent components analysis (ICA) decomposition of the data. Additionally, Step 4 provides the opportunity to load in custom preprocessing code, allowing the user to perform any desired preprocessing steps not typically performed by PSGpower as part of automated batch processing.

### 2.2. Software functionalities

**Fig. 2.**
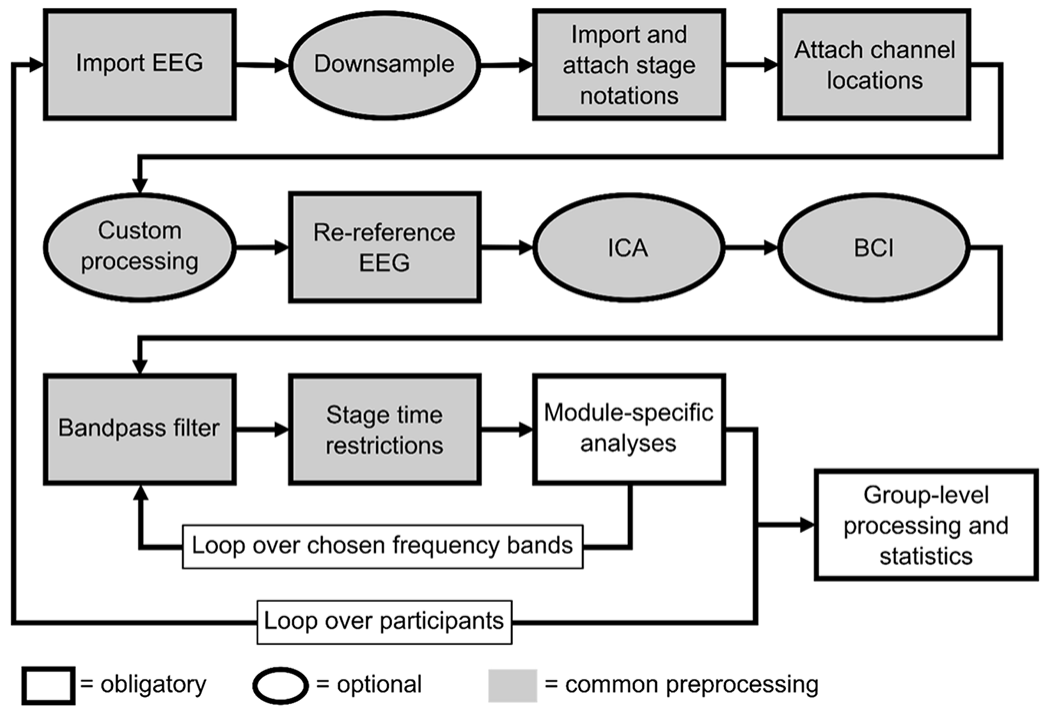
Overall PSGpower structure. Each participant’s data first undergo the common preprocessing steps, indicated here with grey shading. The final two steps of common preprocessing (bandpass filtration and stage time restriction), and all module-specific data processing, are repeated for each frequency band requested in the PSGpower GUI. After all individual participants have been processed, any group-level processing or statistical comparisons included in the chosen analysis module are performed. Details of these steps are summarized in the article text, and fully specified in the PSGpower documentation and open source code.

Once all configuration choices have been made in the GUI, the user clicks the Analyze button to launch the selected analysis module. PSGpower then processes each participant one at a time, starting with a series of module-agnostic data processing steps collectively referred to as the “common preprocessing” (Fig. 2). After the common preprocessing completes, the module-specific data processing and analysis is performed separately for each requested frequency band within each participant. After all participants have been processed individually, module-specific group level processing and analyses are performed, if applicable. For full details regarding the common preprocessing, module-specific individual processing, and module-specific group-level processing and analysis, please see the PSGpower documentation or open source code, which are both distributed with the application. These steps are also summarized in brief here.

The common preprocessing steps are, in order: 1) import EEG data (.edf or .vhdr/.vmrk/.eeg format) into EEGLAB; 2) downsample the EEG if requested; 3) import stage notation metadata in Hume [19], RemLogic (Natus Medical Incorporated, Middleton, WI), or TWin (Grass Technologies, Middleton, WI) format, convert to embedded EEGLAB event markers indicating sleep stage every 30 s, and save to disk in EEGLAB . set/.fdt format; 4) determine and attach channel location information to the EEG; 5) run any custom preprocessing code supplied by the user; 6) re-reference the EEG; 7) run independent components analysis (ICA) on the EEG and save the weights to disk if requested; 8) perform bad channel interpolation (BCI) on the EEG if requested; 9) filter the EEG to the requested frequency band using sleep-EEG-optimized filters; and 10) apply any requested stage time restrictions to the EEG. After these steps are completed, module-specific processing and analysis steps are performed. The filtering, stage time restriction, and module-specific steps are repeated for each requested frequency band before moving on to the next participant (Fig. 2).

The initial release of PSGpower includes seven analysis modules: Hypnogram, Spectra (EEGLAB), Spectra (FieldTrip), Power (Hilbert), Power (newtimef), Spindle Detection (Ferrarelli), and Envelope Viewer. The basic function of each module is transparent from the name: Hypnogram plots the stage time axis (i.e., hypnogram) for a recording, the two Spectra modules allow stage-time aware quantification and comparison of spectral amplitude using the tools built into EEGLAB and FieldTrip, the two Power modules allow stage-time aware quantification and comparison of time-domain amplitude or power using the filter-Hilbert method or wavelet-based time-frequency decomposition, the Spindle Detection module uses an adapted version of an algorithm previously published by Ferrarelli et al. [2] to automatically detect and quantify sleep spindles, and Envelope Viewer plots and quantifies amplitude or power envelopes generated by the Power (Hilbert) module. At the group level, Spectra (EEGLAB) and Spectra (FieldTrip) create and save group-averaged spectral amplitude plots, and Spectra (FieldTrip) can compare spectral amplitude between two groups using cluster-based permutation analysis over the frequency and topography dimensions [15,17]. Envelope Viewer can also compare time-domain amplitude or power using cluster-based permutation analysis over the topography dimension. Note that Envelope Viewer omits the common preprocessing steps, as they have already been performed on the output of Power (Hilbert).

## 3. Illustrative examples

**Fig. 3.**
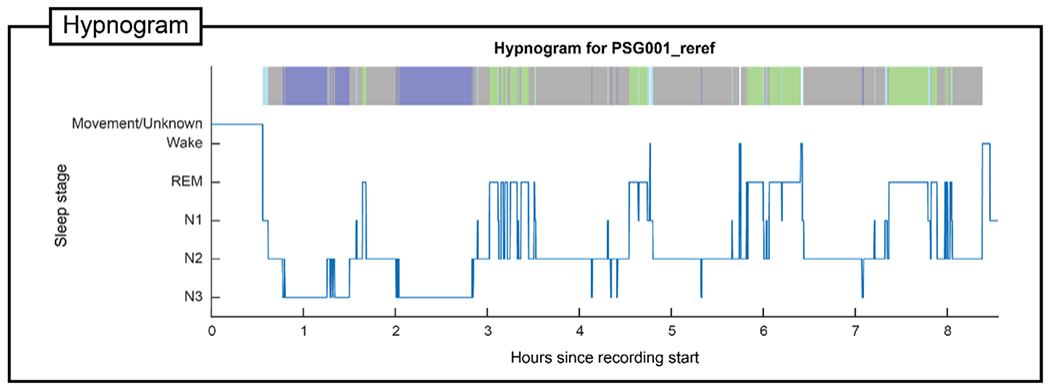
Hypnogram output. Sleep stage metadata are shown in standard hypnogram format for the example young adult overnight recording, with chronological time on the x-axis and sleep stage on the y-axis. Lower stages on the y-axis indicate deeper sleep depth. Sleep stage metadata are also plotted in a more compact ribbon hypnogram format above, with stage indicated by color as follows: cyan = N1, grey = N2, blue = N3, green = REM, white = wake or movement. REM = rapid eye movement; N1 = NREM stage 1; N2 = NREM stage 2; N3 = NREM stage 3.

**Fig. 4.**
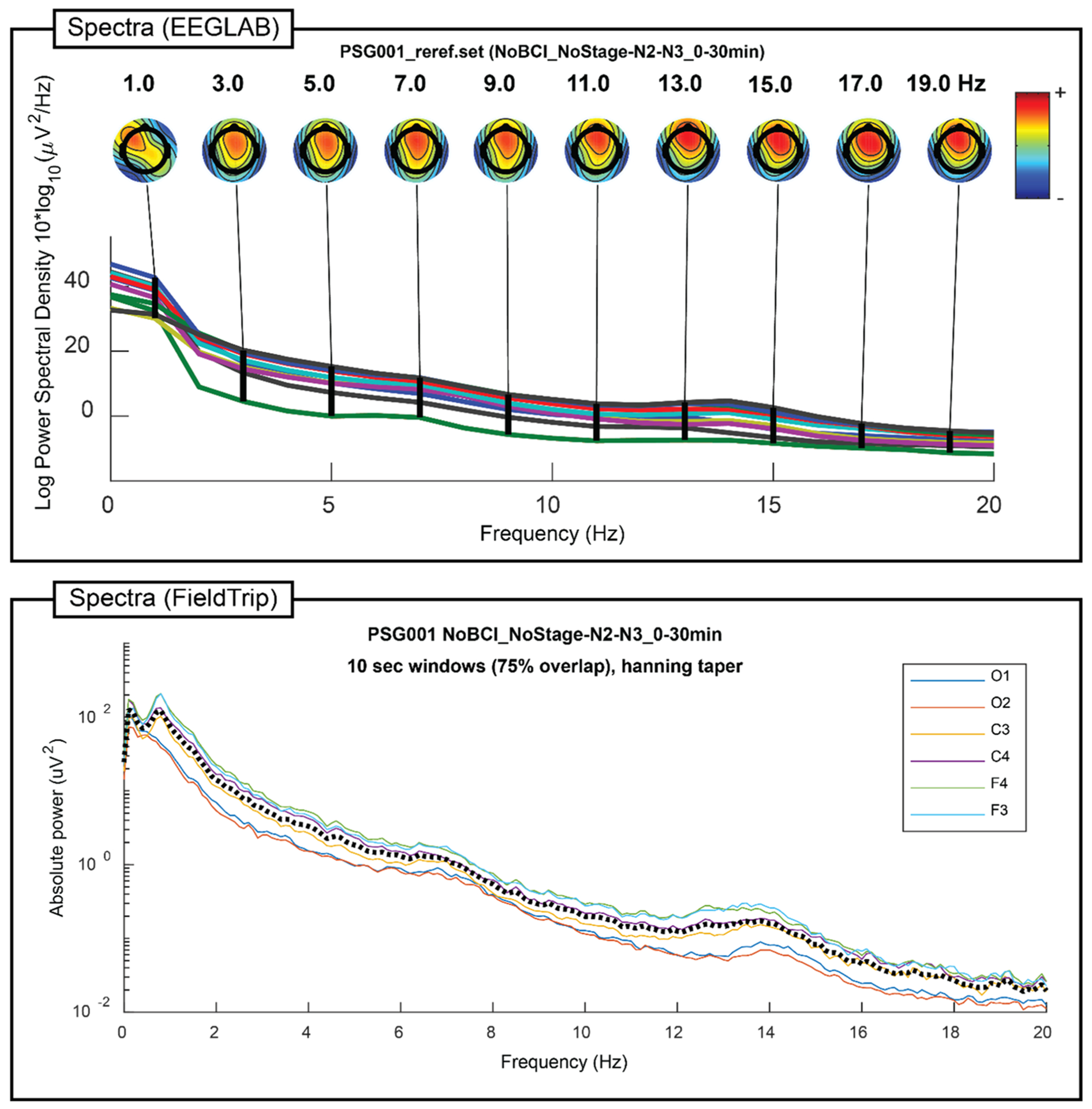
Spectra (EEGLAB) and Spectra (FieldTrip) individual participant output. Frequency-domain amplitude spectra are plotted by electrode for the example young adult overnight recording using the methods built-in to EEGLAB and FieldTrip. Both plots specifically show spectral amplitude only within the first 30 min of either N2 or N3 sleep, demonstrating PSGpower’s capability to flexibly combine stage time and chronological time restrictions for targeted analyses. These plots illustrate the high delta (0.5 – 4 Hz) and sigma (12 – 16 Hz) amplitude typical of sleep EEG, reflecting slow wave and spindle activity respectively.

**Fig. 5.**
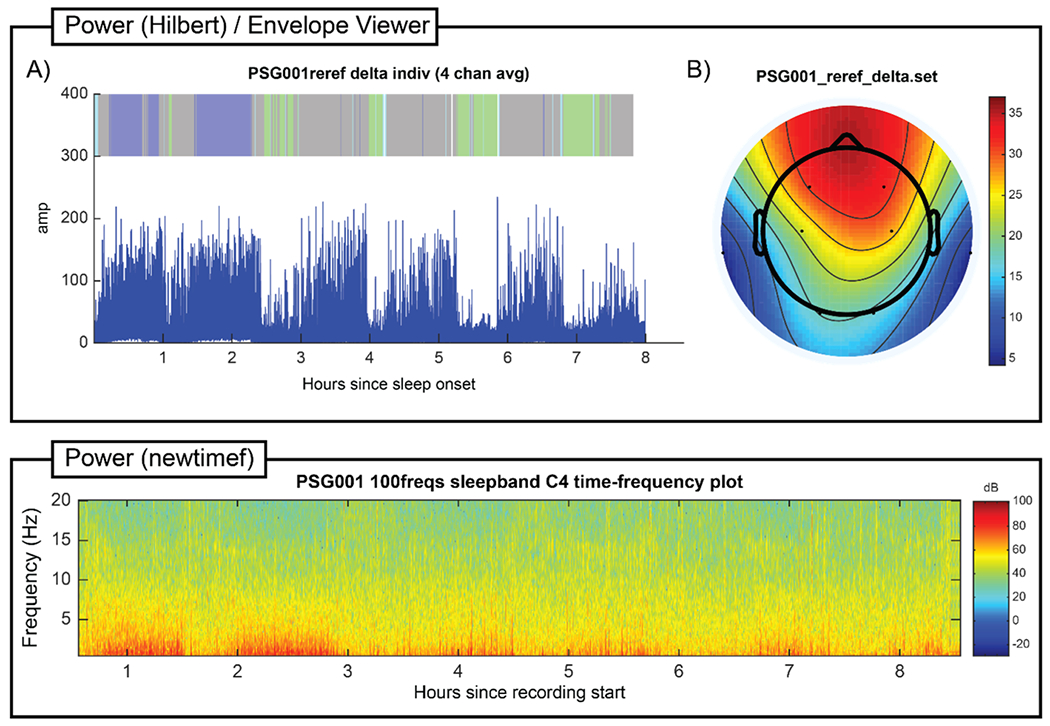
Power (Hilbert) / Envelope Viewer and Power (newtimef) individual participant output. The Envelope Viewer plot shows the delta amplitude envelope generated by Power (Hilbert) for the example young adult overnight recording (A) for the full sleep bout averaged over frontocentral electrodes (F3, F4, C3, C4), and (B) over all electrodes averaged over the first 30 min of N2 or N3 sleep. The electrode-averaged full sleep bout plot includes a ribbon hypnogram over the amplitude envelope for reference, using the color mapping described in the Fig. 3 caption. The Power (newtimef) plot shows the time-frequency representation (TFR) generated for one electrode (C4) of the example young adult overnight recording, with chronological time on the x-axis and frequency on the y-axis; the increased delta (especially in N3) and sigma (especially in N2) band activity characteristic of sleep EEG is apparent, as is the higher delta amplitude typical of early sleep.

**Fig. 6.**
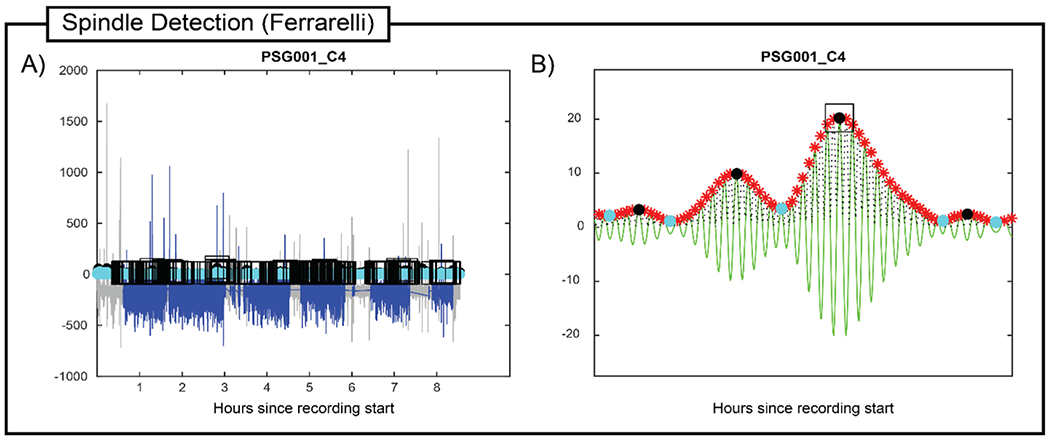
Spindle Detection (Ferrarelli) output. For one electrode (C4) of the example young adult overnight recording, spindles detected by the Ferrarelli algorithm are shown as (A) an overview of all detected spindles (black boxes) relative to chronological time overlaid on the full bandwidth (blue) and sigma-filtered (cyan) EEG, and (B) a zoomed-in view of the sigma-filtered EEG showing one detected spindle. For this detection, the Lower Threshold was set to 2, and the Upper Threshold was set to 6.

Figs. 3, 4, 5, and 6 show the primary graphical output of individual participant processing by the seven modules included in PSGpower for one young adult overnight recording. For full descriptions of all displayed or saved output from each module, please see the documentation. The young adult overnight recording used is taken from a project examining the effects of sleep on memory reconsolidation [9], and is one of the young adult data points included in analyses in this manuscript.

## 4. Impact

The primary impact of PSGpower is making stage-time aware analyses of sleep EEG data simpler to perform. This enables sleep researchers to interrogate information-rich sleep EEG datasets in new ways more rapidly, and expands access to advanced command-line driven EEG tools to sleep researchers who do not have extensive programming experience. We anticipate this will allow sleep research groups to move faster, and potentially expand the use of more involved sleep EEG analysis methods like time-frequency representation analysis and cluster-based permutation analysis. Moreover, the extensibility of PSGpower can facilitate the rapid and stable sharing of “home-grown” sleep EEG analysis methods across research groups, as well as the expansion of existing analysis modules to additional legacy or new sleep EEG or stage metadata formats, which we believe could help the sleep research field as a whole move forward more rapidly and cohesively.

In our internal usage PSGpower has thus far had the desired impact, decreasing the friction of sleep EEG analyses and allowing rapid sharing of methods across research projects. We have used PSGpower as the primary means for analyzing sleep microstructure in eight peer-reviewed manuscripts [4–5,9–11,13,16] and three peer-reviewed published abstracts [3,12,18]. Methodological improvements from each experiment feed back into the software for better results in future work. For example, as part of our older adult work we iterated through several different filter designs to sufficiently isolate theta-band activity from delta-band activity during deep sleep. Nap theta activity isolated using the final Chebyshev II filter design (described first in [5]) showed robust relationships with motor sequence learning over the nap that were not observed for delta or sigma activity, which is an uncommon finding. Because this filter is now implemented as the default theta filter in PSGpower, we can easily apply it in our other work to test for further involvement of theta activity in sleep-dependent memory consolidation that may have been missed with suboptimal filtering.

Another benefit afforded by the structure of PSGpower is a common analysis interface for data from many sources. This benefit was illustrated in our internal usage when the global COVID-19 pandemic caused our work and data collection to become fully remote, including the use of several different portable EEG recording systems. Although each of these portable EEG systems came with their own proprietary software for recording and analysis, once staged and exported to .edf format our researchers were able to analyze these data using the same PSGpower tools they were already familiar with, rather than having to learn multiple new software interfaces. This ability to use familiar analysis tools reduced the productivity costs of adapting to multiple new recording systems. This anecdotal report speaks to the utility of a common analysis framework with extensible input and analysis modules; our intention in releasing PSGpower to the community is that with multiple research groups contributing input and analysis modules around this common framework, methodological gains can be shared more rapidly and overall progress and experimental rigor will increase.

## 5. Conclusions

PSGpower facilitates the flexible analysis of sleep EEG microstructure by considering sleep stage metadata throughout EEG processing and analyses, in EEGLAB-based, FieldTrip-based, and custom workflows. The structure of PSGpower is modular and extensible, allowing the addition and sharing of custom analysis workflows within and across labs, and the application of existing analysis workflows to new EEG and sleep stage data formats as needed. This common framework approach has improved the sharing, speed, and reliability of sleep EEG microstructure analyses in our laboratory, and we are releasing these tools publicly because we believe these improvements will scale to the wider sleep EEG research community with broader use.

## Data Availability

Data will be made available on request.
